# Quality of life in caregivers of a child with a developmental and epileptic encephalopathy

**DOI:** 10.1111/dmcn.15695

**Published:** 2023-07-08

**Authors:** Eden G. Robertson, Lauren Kelada, Stephanie Best, Ilias Goranitis, Kristine Pierce, CoGENeS Group, Natalie J Roberts, Rani Sachdev, Fleur Le Marne, Rebecca Macintosh, Erin Beavis, Annie Bye, Elizabeth E. Palmer

**Affiliations:** ^1^ Discipline of Paediatrics and Child Health, School of Clinical Medicine UNSW Medicine & Health, UNSW Sydney Randwick Australia; ^2^ Behavioural Sciences Unit, Kids Cancer Centre Sydney Children's Hospital Randwick Australia; ^3^ Department of Health Services Research Peter MacCallum Cancer Centre Melbourne Australia; ^4^ Victorian Comprehensive Cancer Centre Melbourne Australia; ^5^ Sir Peter MacCallum Cancer Centre Dept of Oncology University of Melbourne Melbourne Australia; ^6^ Australian Genomics Health Alliance Murdoch Children's Research Institute Melbourne Australia; ^7^ Health Economics Unit, Centre for Health Policy, Melbourne School of Population and Global Health University of Melbourne Melbourne Australia; ^8^ Epilepsy Foundation Surrey Hills Melbourne Australia; ^9^ Centre for Clinical Genetics Sydney Children's Hospitals Network ‐ Randwick Randwick Australia; ^10^ Department of Neurology Sydney Children's Hospitals Network ‐ Randwick Randwick Australia

## Abstract

**Aim:**

To explore the relationship between social care‐related quality of life (SCrQoL) for caregivers of a child with a developmental and epileptic encephalopathy (DEE; such as SCN2A and Dravet syndrome) and health literacy, illness perceptions, and caregiver activation.

**Method:**

As part of a larger pre‐post pilot study of an information linker service, caregivers completed a baseline questionnaire which included demographics and measures to assess SCrQoL, health literacy, illness perceptions, and caregiver activation. We used Spearman's Rho to determine relationships between variables.

**Results:**

Seventy‐two caregivers completed the questionnaire. Total SCrQoL varied widely, ranging from an ‘ideal state’ to ‘high needs state’. Caregivers most frequently reported high needs regarding doing activities they enjoy and looking after themselves. Total SCrQoL was correlated with cognitive (r[70] = −0.414, *p* < 0.000) and emotional representations of illness (r[70] = −0.503, *p* < 0.000), but not coherence (r = −0.075, *p* = 0.529). Total SCrQoL was not correlated with health literacy (r[70] = 0.125, *p* = 0.295) or caregiver activation (r[70] = 0.181, *p* = 0.127).

**Interpretation:**

Future research should explore whether interventions that help caregivers cognitively reframe the negative experiences of having a child with a DEE, and support them to partake in activities they enjoy, boost their SCrQoL.

**What this paper adds:**

- Caregiver social care‐related quality of life (SCrQoL) varied widely, from ‘ideal state’ to ‘high needs state’.
- Most common high needs were doing enjoyable activities and self‐care.
- Caregivers with higher SCrQoL may perceive their child's illness as less threatening.
- SCrQoL does not appear to be related to caregiver activation in this sample.

## What this paper adds

This original article is commented on by Chen on page 143 of this issue.

Video Podcast: https://youtu.be/‐tjFkkJyJY8

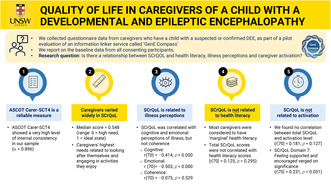

**Abbreviations**
ASCOT Carer–SCT4: Adult Social Care Outcomes Toolkit–four‐level self‐completion questionnaire for carers
DEE: developmental and epileptic encephalopathy
QoL: quality of life
SCrQoL: social care‐related quality of life

Developmental and epileptic encephalopathies (DEEs) are the most severe group of epilepsies with high mortality and profound morbidity. Onset is usually in infancy or childhood with treatment‐resistant seizures, epileptiform activity on electroencephalogram, developmental slowing or regression, and cognitive impairment. Combined, the incidence of DEEs is more than 1 in 2000 live births.^1^ However, they are highly genetically heterogeneous with over 400 monogenic causes,^2^ meaning each individual genetic DEE diagnosis is extremely rare.^3^ Significant improvements in genomic techniques and access to this technology means more children now receive a genetic diagnosis.^1^, ^2^, ^3^

Caregivers of a child with a rare disease are at risk for poorer quality of life (QoL) and wellbeing compared to the general population.^4^ For caregivers who have a child with DEE specifically, there is mounting research demonstrating these complex conditions have a negative impact on caregivers' QoL and wellbeing.^5^, ^6^ They may also experience greater psychological distress than caregivers of children with other rare diseases, such as cystic fibrosis^7^ and other childhood‐onset neurogenetic conditions.^8^

Social care‐related QoL (SCrQoL) refers to QoL in the context of caring for an individual, such as a child. This concept captures a more holistic appreciation of an individual's QoL (e.g. a caregiver's QoL), beyond their health status.^5^ Improved understanding of caregiver SCrQoL and factors that may contribute towards better outcomes are needed to develop appropriate resources and support. Modifiable factors of caregivers' experience, such as subjective measure of seizure burden,^6^ emotional and financial stress,^9^ and amount of social support can impact caregiver wellbeing beyond more objective and disease‐specific predictors (e.g. number of seizures).^4^, ^10^ Research highlights that parents of a child with early‐onset epilepsy have unmet information needs, associated with greater levels of stress and poorer psychosocial outcomes.^11^ Such unmet information needs may be due to poorer health literacy (i.e. how people access, understand, and use health information), as seen across other health conditions, which is related to poorer QoL.^12^ Perceived understanding of the illness, in addition to other illness perceptions (such as perceived personal control) have also been shown to relate to anxiety and depression symptoms for people caring for a child with a neurological condition.^13^

Given the chronicity and severity of DEEs, it is integral that caregivers are supported in managing their child's health care. Improving patient activation (i.e. the knowledge, skills, and confidence in dealing with the range of tasks and challenges inherent in disease management) has been shown to significantly improve patients' psychosocial health.^14^ This suggests the potential value of strengthening activation in caregivers who have a child with a DEE. Increasing caregivers' capacity for active participation in their child's care not only has implications for improving their own wellbeing, but potentially improving QoL for other families living with a rare disease.^15^

To provide insight into a group of vulnerable caregivers and identify areas for potential intervention, we explored the relationship between SCrQoL, health literacy, illness perceptions, and caregiver activation.

## METHOD

We collected survey data as part of a larger pilot study evaluating the acceptability and feasibility of ‘GenE Compass’, an information linker service for caregivers of a child with a DEE. Caregivers submit questions about their child's condition to our expert multidisciplinary team via an online form and receive a personalized report via e‐mail. The aim of GenE Compass is to provide high‐quality, relevant, and understandable information so that caregivers: (1) feel informed, (2) feel empowered to be more involved in health care discussions/decisions, and (3) spend less time seeking information, often seen within the rare disease space.^16^ The pilot study involved baseline data collection (survey) and follow‐up after 3 months of access to GenE Compass (survey and interview). The protocol for this evaluation is published^17^ and registered on the Australian New Zealand Clinical Trials Registry (ACTRN12621001544864). We received ethics approval from the Sydney Children's Hospitals Network Human Research Ethics Committee (2021/ETH11277).

### Participants

We invited caregivers (e.g. mother, father, grandparent) to participate in GenE Compass if they: (1) had a child (<18 years of age at time of study invitation) with a clinically suspected or confirmed diagnosis of DEE, and (2) spoke/read English sufficiently to be able to complete the questionnaires. More than one caregiver from the same family could participate, however this did not occur.

### Recruitment

Between January 2022 and June 2022, we actively recruited all primary caregivers (as indicated in the child's hospital medical records) with a child cared for within the Sydney Children's Hospitals Network, and meeting the eligibility criteria. We contacted families via e‐mail or mail, and invited any caregiver to participate. We followed up a maximum of three times.

### Measures

We report on data collected by a baseline questionnaire from the evaluation of GenE Compass. This questionnaire captured demographics and SCrQoL (Adult Social Care Outcomes Toolkit–four‐level self‐completion questionnaire for carers [ASCOT Carer–SCT4]),^18^, ^19^ health literacy (BRIEF: Health Literacy Screening Tool),^20^, ^21^ illness perceptions (Brief Illness Perception Questionnaire),^22^, ^23^ and caregiver activation (Patient Activation Measure–short form, adapted).^24^, ^25^ No outcome data were missing as all items required a response. See Table 1 for an overview of measures.

**TABLE 1:** Overview of measures

Construct	Measure	Psychometric properties	Description of measure	Scoring
Social care‐related quality of life	Adult Social Care Outcomes Toolkit–four‐level self‐completion questionnaire for carers (ASCOT Carer–SCT4)^18^, ^19^	Demonstrated strong psychometrics in a sample of Australian informal carers (e.g. parent) across a range of conditions (e.g. chronic disease or disability)^18^, ^19^	An 8‐item self‐reported measure to assess the impact of social care support on unpaid carers' quality of life. Each item of the measure represents a single domain: Occupation Control over daily life Looking after yourself Personal safety Social participation and involvement Space and time to be yourself Feeling supported and encouraged	Each domain is rated on a 4‐level scale, ranging from the ideal state (level 1) to high needs (level 4). Total weighted scores range from 0 (high level needs: ‘where there are needs, and these have an immediate or longer‐term health implication’) to 1 (ideal state: ‘the individual's needs are met to their preferred level’)
Health literacy	Brief Health Literacy Screening Tool	Validated in adults in the USA, with high sensitivity^20^, ^21^	A 4‐item self‐reported measure to determine the degree to which one can read, understand, exchange, and use health information and resources	Total score range: 4 (low health literacy) to 20 (high health literacy) Limited (4–12) Marginal (13–16) Adequate (17–20)
Perceptions of illness	Brief Illness Perception Questionnaire	Widely used measure across several illnesses, with strong psychometrics across a range of cohorts^22^, ^23^	A 9‐item measure designed to assess: Cognitive representation of illness, calculated by sum of the following items: consequences timeline personal control treatment control identity Emotional representation of illness, calculated by sum of the following items: concern about illness effect on mood Coherence/understanding Perceived cause of illness: this item was not asked as it does not contribute to the overall score	Representation of illness range: 0 (less negative perspectives of illness) to 50 (more negative perspectives of illness) Emotional representation of illness range: 0 (no concern/emotional impact) to 20 (greater concern/emotional impact) Comprehensibility/understanding range: 0 to 10
Caregiver health care activation	Patient Activation Measure–short form, adapted	Original version shown to be reliable and valid in adults patients,^24^ including adult patients with a neurological condition^25^	A 13‐item measure that assesses knowledge, skill, and confidence for health management, adapted for caregivers to respond to items regarding their child's health	Total raw scores = sum of responses to each item. Possible total scores ranged from 13 (low activation) to 52 (high activation)

### Statistical analysis

We analysed data using SPSS Statistics, version 26 (IBM Corp., Armonk, NY, USA)^26^ and used descriptive statistics to describe demographics and outcome variables. We calculated total scores for outcomes following developer instructions. For the SCrQoL, certain attributes of carers' SCrQoL are likely to be more important than others. To account for this in producing a single overall SCrQoL score, we used Batchelder et al.'s preference‐based index values for each of the measures' domains. This allowed us to assign a preference‐based index values based on participants' response to each domain, and sum these index values to obtain a single total score which could range from 0 (high‐level needs) to 1 (ideal state).^18^

For caregiver activation, we calculated total raw scores by summing the responses to each item. Possible total scores ranged from 13 (low activation) to 52 (high activation),^24^ with responses of ‘not applicable’ treated as missing. For health literacy, we summed responses to each item to obtain a total score. Possible total health literacy scores ranged in value from 4 (inadequate health literacy) to 20 (adequate health literacy).^20^ For illness perceptions, there are three domains: ‘cognitive representations of illness’; ‘emotional representations of illness’; and ‘coherence’. We calculated each domain by aggregating responses to relevant questions, with reverse scoring as necessary. Higher scores represent a more threatening view of illness.^22^

We ran a Cronbach's alpha test to assess internal reliability of the ASCOT Carer–SCT4 total index scores in our sample, as advisable to measure each time a test is administered.^27^ As total index scores showed a skew to the right, we used relevant Spearman's Rho to determine the relationship between SCrQoL scores (total and for each domain), and illness perceptions (cognitive representations, emotional representations, and coherence), health literacy, and caregiver activation. Significance was defined a priori as a *p*‐value lower than 0.05, two tailed.

## RESULTS

We invited 168 eligible families to participate in GenE Compass from January to June 2022, with recruitment closing because of funding limitations. Of these, 72 caregivers provided written informed consent, of whom 68 completed the questionnaire (response rate = 68/168, 40.5%). We did not capture reasons for non‐participation for ethical reasons. Our research team were approached by four caregivers who heard about our study at a family conference in July 2022, via social media or through their neurologist after a recent genetic diagnosis. These four caregivers participated in our study, with 72 caregivers in total completing the baseline questionnaire (Figure S1).

### Demographics

Caregivers were 39 years 10 months old on average, with most being highly educated, mothers, and living in a metropolitan area. At questionnaire completion, children of participating caregivers were 8 years 4 months old on average. Most children in our study had received a genetic diagnosis (*n* = 48, 66.7%), representing a wide range of individual genetic diagnoses (Table 2).

**TABLE 2:** Demographics of caregivers and children

Parent demographics (*n* = 72)	Mean (SD) / *n* (%)
Parent age (years:months) at survey completion (missing = 1)	39:10 (8:10)
Mother	58 (80.6%)
Father	14 (19.4%)
Highest education	
Year 11 or below	2 (2.8%)
Year 12	4 (5.6%)
Apprenticeship	1 (1.4%)
Certificate/Diploma	25 (34.7%)
University degree	21 (29.2%)
Postgraduate degree	19 (26.4%)
Employment	
Full‐time	23 (31.9%)
Part‐time	23 (31.9%)
Casual	6 (8.3%)
Not employed	
Home duties	15 (20.8%)
Not seeking work/retired	5 (6.9%)
Number of children with DEE	
1 child	70 (95.9%)
2 children	3 (4.1%)
Marital status	
Currently married or de facto	61 (84.7%)
Separated/divorced/previously de factor	9 (12.5)
Never married or de facto	2 (2.8%)

Child demographics (*n* = 75)	Mean (SD) / *n* (%)
Child age (years:months) at survey completion (missing = 2)	8:4 (4:4)
Primary neurologist team (missing = 3, 4%)	
Sydney Children's Hospital Randwick	48 (64.0%)
The Children's Hospital at Westmead	24 (32.0%)
Diagnosis^a^	
DEE	42 (58.3%)
Genetic aetiology specified^b^	33
Genetic aetiology not specified	9
Dravet syndrome (SCN1A‐related condition)	7 (9.7%)
Early infantile DEE	10 (13.9%)
Genetic aetiology specified^c^	4
Genetic aetiology not specified	6
Epilepsy with eyelid myoclonia (genetic aetiology specified)	1 (1.4%)
Epilepsy with myoclonic‐atonic seizures	5 (6.9%)
Genetic aetiology specified^c^	2
Genetic aetiology not specified	3
Febrile infection‐related epilepsy syndrome	1 (1.4%)
Lennox–Gastaut syndrome	2 (2.8%)
Genetic aetiology specified^c^	1
Genetic aetiology not specified	1
Infantile epileptic spasms syndrome (genetic aetiology not specified)	4 (5.6%)

^a^ Diagnosis categories based on International League Against Epilepsy classification and definitions.

^b^ Individual genetic diagnoses are not listed because of the potential identifying nature, however, are inclusive of genes such as *UBE3A, KCNQ2, DNM1, CHD2*, and *GABRA1*.

^c^ Specific genetic aetiologies are not shared because of data being highly identifiable.

Abbreviation: DEE, developmental and epileptic encephalopathy.

### SCrQoL in caregivers of a child with a DEE

**FIGURE 1:**
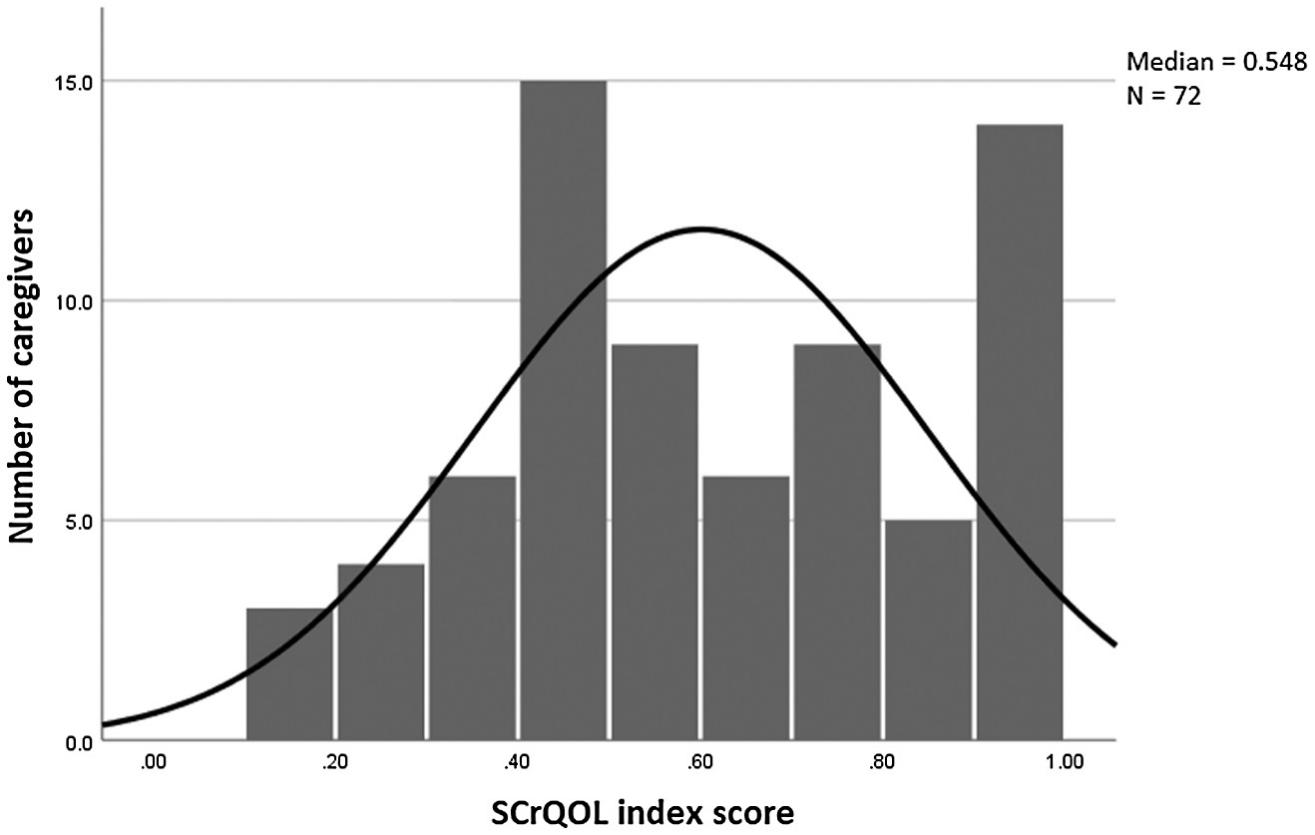
Histogram of caregiver social care‐related quality of life (SCrQoL) using the Adult Social Care Outcomes Toolkit–four‐level self‐completion questionnaire for carers (*n* = 72).

Examination of a histogram and Q–Q plot showed total SCrQoL as having a slight skew (see Figure 1). The median total SCrQoL score was 0.548, with caregivers' scores ranging from 0 (high need) to 1 (ideal state) (α = 0.896). Of the seven SCrQoL domains, caregivers most commonly rated some needs or high‐level needs for ‘Domain 3: Looking after yourself’ (such as getting enough sleep or eating well), with 39 caregivers (54.2%) rating some need or high‐level need; and ‘Domain 1: Occupation’ (that is, doing activities that they enjoy or value), with 56 caregivers (77.8%) rating some needs or high‐level needs. Most caregivers self‐reported no needs regarding ‘Domain 4: Personal safety’, with 65 caregivers (90.3%) indicating no or few needs (Table 3).

**TABLE 3:** Frequency of responses across each SCrQoL domain and illness perceptions (*n* = 72)

	Frequency of responses, *n* (%)			
Domains of SCrQoL (via the ASCOT Carer–SCT4)	Ideal state	No needs	Some needs	High level needs
Domain 1: Occupation (I'm able to spend my time as I want, doing things I value or enjoy)	4 (5.5%)	12 (16.4%)	49 (67.1%)	7 (9.6%)
Domain 2: Control over daily life (I have as much control over my daily life as I want)	8 (11%)	23 (31.5%)	35 (47.9%)	6 (8.2%)
Domain 3: Looking after yourself (I look after myself as well as I want)	5 (6.8%)	28 (38.4%)	21 (28.5)	18 (24.7%)
Domain 4: Personal safety (I feel as safe as I want)	49 (67.1%)	16 (21.9%)	4 (5.5%)	3 (4.1%)
Domain 5: Social participation and involvement (I have as much social contact as I want)	11 (15.1%)	21 (28.8%)	27 (37.0%)	13 (18.1%)
Domain 6: Space and time to be yourself (I have all the space and time I need to be myself)	9 (12.3%)	13 (17.8%)	39 (53.4%)	11 (15.1%)
Domain 7: Feeling supported and encouraged (I feel I have the encouragement and support I want)	13 (17.8%)	23 (31.5%)	34 (46.6%)	2 (2.7%)

**Domains of illness perceptions (via the Brief‐ Illness Perceptions Questionnaire)**	**Median (range)**			
**Cognitive representations (median = 36.0; 8–49)**				
Consequences (How much does your child's illness affect your life?)	8.5 (1–10)			
Timeline (How long do you think your child's illness/symptoms will continue?)	10 (0–10)			
Personal control (How much control do you feel you have over your child's illness/ symptoms?)	7 (0–10)			
Treatment control (How much do you think your child's treatment can help his/her illness/ symptoms?)	4 (0–10)			
Identity (How much does your child experience symptoms from his/her illness?)	8 (0–10)			
**Emotional representations (median = 17.0; 5–20)**				
Concern (How concerned are you about your child's illness?)	0 (0–10)			
Effect on mood (How much does your child's illness affect you emotionally?)	8 (2–10)			
**Coherence**				
Coherence (How well do you feel you understand your child's illness?)	3 (0–10)			

Abbreviations: ASCOT Carer‐SCT4, Adult Social Care Outcomes Toolkit–four‐level self‐completion questionnaire for carers; SCrQoL, social care‐related quality of life.

### SCrQoL is associated with illness perceptions

The median total score on the Brief Illness Perceptions Questionnaire was 56.0, with scores ranging from 17 (a less threatening view of illness) to 74 (a more threatening view of illness). For cognitive representation of illness, the median score was 36.0 (range = 8–49); for emotional representation of illness, the median score was 17.0 (range = 5–20); and for coherence, the median score was 3.0 (range = 0–10) (Table 3).

Total SCrQoL was correlated with cognitive representations (r[70] = −0.414, *p* < 0.000) and emotional representations of illness (r[70] = −0.503, *p* < 0.000), but not coherence (r = −0.075, *p* = 0.529). When exploring the individual domains of SCrQoL, we found that cognitive representation scores were correlated with all domains except ‘Domain 4: Personal safety’. Emotional representation scores were correlated for all domains (*p* < 0.05). Coherence was not correlated with any domain (Table 4).

**TABLE 4:** Non‐parametric correlations between SCrQoL and health literacy, activation, and illness perceptions (*n* = 72)

	Total SCrQoL	1: Occupation	2: Control over daily life	3: Looking after yourself	4: Personal safety	5: Social participation and involvement	6: Space and time to be yourself	7: Feeling supported and encouraged
**Total illness perceptions**	r = −0.484, *p* < 0.000^a^	r = −0.410, *p* < 0.000^a^	r = −0.516, *p* < 0.00^a^	r = −0.405, *p* < 0.000^a^	r = −0.259, *p* = 0.028^a^	r = −0.484, *p* < 0.000^a^	r = −0.390, *p* < 0.001^a^	r = −0.359, *p* = 0.001^a^
**Cognitive representations of illness**	r = −0.414, *p* < 0.000^a^	r = −0.392, *p* < 0.001^a^	r = −0.460, *p* < 0.000^a^	r = −0.308, *p* = 0.009^a^	r = −0.165, *p* = 0.166	r = −0.443, *p* = 0.000^a^	r = −0.331, *p* < 0.004^a^	r = −0.311, *p* = 0.008^a^
**Emotional representations of illness**	r = −0.503, *p* < 0.000^a^	r = −0.290, *p* = 0.014^a^	r = −0.487, *p* < 0.000^a^	r = −0.509, *p* < 0.000^a^	r = −0.340, *p* = 0.004^a^	r = −0.404, *p* < 0.000^a^	r = −0.467, *p* < 0.000^a^	r = −0.378, *p* = 0.001^a^
**Coherence**	r = −0.075, *p* = 0.529	r = −0.121, *p* = 0.313	r = −0.123, *p* = 0.302	r = −0.018, *p* = 0.879	r = −0.159, *p* = 0.183	r = 0.078, *p* = 0.513	r = 0.022, *p* = 0.853	r = 0.114, *p* = 0.339
**Health literacy**	r = 0.125, *p* = 0.295	r = 0.217, *p* = 0.067	r = 0.110, *p* = 0.357	r = 0.079, *p* = 0.512	r = 0.183 *p* = 0.123	r = 0.104, *p* = 0.383	r = 0.106, *p* = 0.376	r = 0.171, *p* = 0.152
**Caregiver activation**	r = 0.181, *p* = 0.127	r = 0.149, *p* = 0.213	r = 0.187, *p* = 0.116	r = 0.149, *p* = 0.211	r = 0.171, *p* = 0.151	r = 0.209, *p* = 0.078	r = 0.131, *p* = 0.273	r = 0.231, *p* = 0.051

^a^ *p* <0.05, two tailed.

Abbreviation: SCrQoL, social care‐related quality of life.

### SCrQoL is not associated with health literacy

The median score on the BRIEF: Health Literacy Screening Tool was 15.0, with scores ranging from 10 (defined as ‘limited’ health literacy) to 20 (defined as ‘adequate’ health literacy). Most caregivers (*n* = 57, 79.2%) were considered as having ‘marginal’ health literacy. Total SCrQoL was not correlated with health literacy (r[70] = 0.125, *p* = 0.295), nor any individual domains of SCrQoL (Table 4).

### SCrQoL is not associated with activation

The median score on the total Patient Activation Measure score was 43.0 (range = 31–52). We found no correlation between total SCrQoL and activation (r[70] = 0.181, *p* = 0.127), nor any correlation between individual domains of SCrQoL and activation. However, ‘Domain 7: Feeling supported and encouraged’ verged on being significant (r[70] = 0.231, *p* = 0.051).

## DISCUSSION

We assessed SCrQoL of caregivers who have a child with a DEE, and its relationship with health literacy, illness perceptions, and caregiver activation. We found that better SCrQoL was associated with a less threatening perception of their child's illness. Interestingly, ‘coherence’ within illness perceptions and health literacy was not associated with any domains of the ASCOT Carer–SCT4, nor was there any association between SCrQoL and caregiver activation.

We found that caregivers reported a wide range of total SCrQoL scores when using the ASCOT Carer–SCT4. In comparison to other informal carers in Australia^28^ and carers of adults in England, our cohort appears to show a higher level of needs. In particular, we found that caregivers had a higher level of needs regarding looking after themselves and engaging in usual activities. Our sample of caregivers across a range of DEEs report higher needs regarding looking after themselves and engaging in usual activities than previous studies that only report on caregivers of a child with the DEE Dravet syndrome.^29^ This is despite 10% of our sample having a child with Dravet syndrome. Other studies focusing on parents of children with DEE or similar severe childhood‐onset epilepsy cohorts but using alternative measures (e.g. EQ‐5D‐5L) show comparable variability of SCrQoL.^9^, ^30^ This indicates that while suspected or confirmed DEE puts caregivers at higher risk of poor SCrQoL, it may not cause poor SCrQoL. Until widely accessible and curative treatments are found, our findings suggest we can learn from how some caregivers cope better with their child's condition, and ultimately intervene using a strengths‐based approach. Alongside logistical and social support for families, supporting caregivers to build resilience and self‐compassion may promote more adaptive coping to reframe negative illness perceptions.^31^

Overall, we found that better SCrQoL was associated with less threatening perceptions of illness. Similar to parents of children with neurological disorders in India,^13^ our cohort reported strong negative illness perceptions, specifically greater concern about their child's illness and impact of symptoms, and high emotional reactions to their child's illness. However, in comparison, our cohort reported more negative perceptions regarding ‘timeline’ and benefit of treatment. Furthermore, we did not find SCrQoL to be related to personal control, treatment control, and coherence/understandability, which contrasts with findings in the Indian parents.^13^ These findings are not unexpected—likely reflecting the life‐threatening and long‐term nature of DEEs, and lack of curative treatment. We also found caregivers' cognitive and emotional representations of illness were highly negatively correlated with their SCrQoL, suggesting a need to better support caregivers to strengthen their emotional stability, resilience, and self‐regulation.

The lack of association between coherence/understandability and SCrQoL aligns with the lack of association between SCrQoL and health literacy. However, previous literature with caregivers looking after someone with a chronic illness suggests otherwise. Specifically, adequate question‐asking health literacy appears associated with lower anxiety and higher QoL for caregivers.^32^ Most participants in our study had a high level of activation and health literacy in comparison to Australian population norms.^33^ This may have limited the generalizability of our findings. Previous research specifically with caregivers of a child with a DEE suggest that they struggle to find reliable, relevant, and understandable information, and this can increase distress and result in poorer QoL.^11^ However, these unmet information needs may be due to a lack of available resources, rather than a perceived inability to understand information provided or confidence in using information to care for their child. A more granular exploration of health literacy in rare disease caregivers is warranted given the perceived lack of accessible information and complexity of medical information these families often receive. Feasible health literacy interventions to support caregivers in finding the information they need, when they need it, is warranted in the era of rapid clinical and genomic advancements and unregulated health information.

To our knowledge, little research to date explores caregivers' health activation in seeking help for their child, and the impact on caregiver QoL. While hypothesized to be related, we did not find any association between SCrQoL and activation. Given the literature shows a strong relationship between patient activation and a broad range of health‐related outcomes, we maintain the importance for caregivers, especially those from lower socioeconomic backgrounds, to be supported to take action in their child's health journey.^34^ Previous studies using derivatives of the Patient Activation Measure have reported a smaller proportion of caregivers in the high activation category, compared to what we found in our sample.^35^, ^36^ Our sample may have particularly high levels of activation because of the severe and refractory nature of DEEs. It is also possible that caregivers who choose to participate in the GenE Compass evaluation study are more likely to have high activation. More research is needed to understand parental activation, and how we can foster and sustain this elevated level of activation.

### Strengths and limitations

To our knowledge, ours is one of few studies within the Australian context that explores SCrQoL of caregivers of a child with a DEE. However, it is not without limitations. First, we collected this data as part of a larger study to evaluate an information linker service. The eligibility criteria for this study limited participation to caregivers of English‐speaking background from two Sydney hospitals, which are part of the largest tertiary children's hospitals network in Australia. This may have resulted in biased responses. While we tried to recruit all eligible families from these two hospitals, the lack of a comprehensive database of all patients with DEEs meant some families may have been missed.

While our sample was sufficient to look at results across all DEEs combined, we lacked the sample size to examine differences based on type of DEE, severity of condition, and impact of having more than one child with a DEE. Exploration of the latter factors, because of the individual rarity of each DEE, will likely require national if not international approaches such as large natural history studies.^37^ As is common in many psychosocial research studies,^38^, ^39^ our sample was predominantly highly educated mothers from metropolitan areas despite several recruitment strategies (e.g. working closely with patient support groups, provision of multimedia resources and infographic postcards). Given the associations between education, socioeconomic status, and health literacy, our findings may be skewed. Most of our sample had high health literacy and activation, which limited our ability to conduct further between‐group comparisons. Finally, our study was also limited by chosen measures. While the BRIEF: Health Literacy Screening Tool was short and reduced burden on study participants, this measure is typically used to determine whether an individual has ‘adequate’ health literacy or not. This lowered sensitivity, and a more detailed measure may be helpful for future studies.

### Conclusion

Our study suggests that SCrQoL in caregivers who have a child with a DEE varies widely. SCrQoL was related to caregivers' perception of how threatening their child's illness is and may not be related to caregiver activation or health literacy as hypothesized. This suggests interventions that target the negative emotional experiences of having a child with a DEE and reframe their situation may enhance caregiver QoL, alongside other clinical and psychosocial supports.

## Supporting information


**Figure S1:** Recruitment flowchart.

## Data Availability

Data is not publicly available because of ethical restrictions. However, data is available upon reasonable request to the corresponding author.
