# Supplementary figures and images for: Quality of life in caregivers of a child with a developmental and epileptic encephalopathy
Source: Dev Med Child Neurol. 2023 Jul 8;66(2):206–15. doi: 10.1111/dmcn.15695 (PMC10952662; doi:10.1111/dmcn.15695)

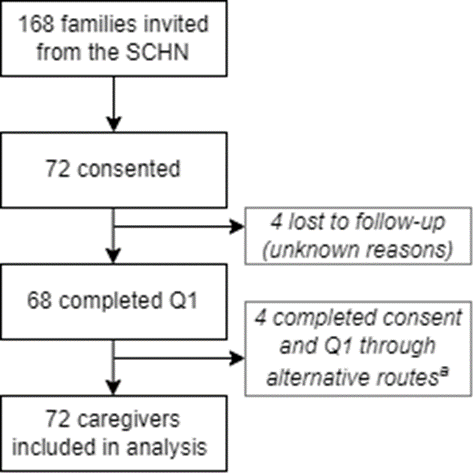

Supplement: Supplementary file 1 — Figure S1: Recruitment flowchart. [file DMCN-66-206-s001.png]
